# Supplementary material for: In-Lipid Structure of Pressure-Sensitive Domains Hints Mechanosensitive Channel Functional Diversity
Source: Biophys J. 2020 Jun 23;119(2):448–59. doi: 10.1016/j.bpj.2020.06.012 (PMC7376121; doi:10.1016/j.bpj.2020.06.012)
Supplement: Document S1. Figs S1–S4 and Tables S1–S3 [file mmc1.pdf]

Biophysical Journal, Volume *119*

Supplemental Information

In-Lipid Structure of Pressure-Sensitive Domains Hints Mechanosensitive Channel Functional Diversity

Charalampos Kapsalis, Yue Ma, Bela E. Bode, and Christos Pliotas

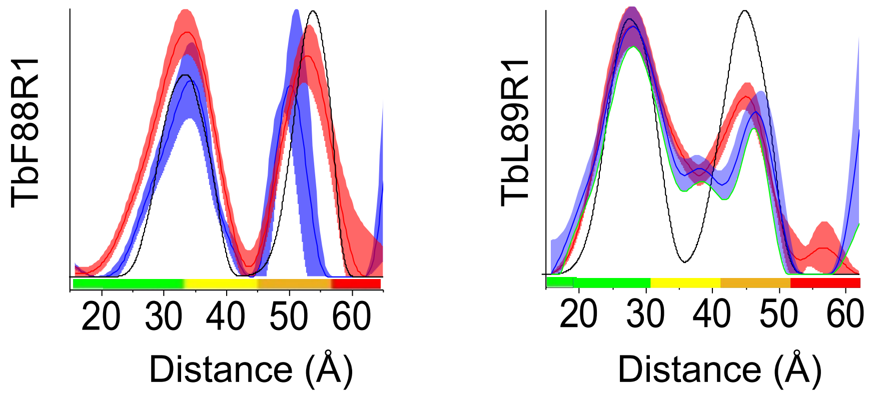

Fig. S1: Liposome (*E coli* polar extract) (shaded blue) and NDs (DMPC) (shaded red) PELDOR distance distributions compared to the modelled distances simulated from the x-ray (closed state) crystal structure (PDB 2OAR) using MtsslWizard (black line) for TbMscL F88R1 and L89R1.

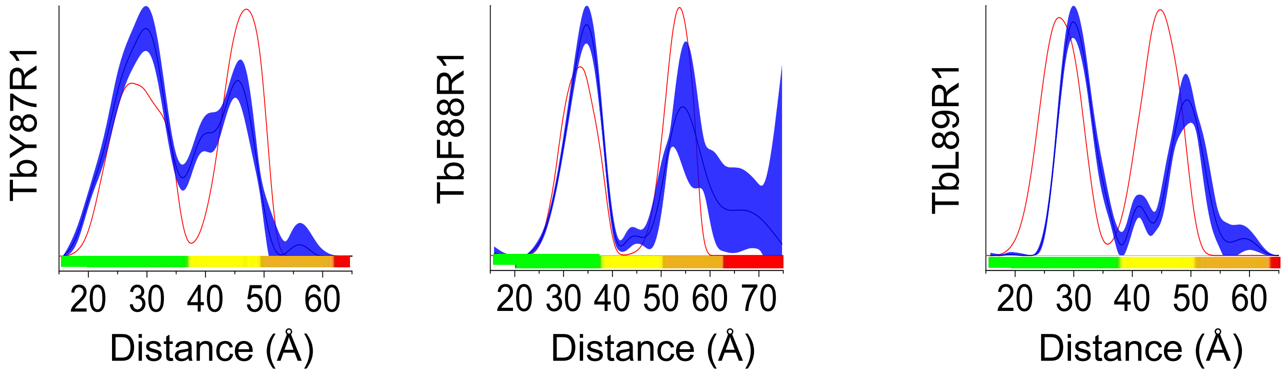

Fig. S2: PELDOR distance distributions of TbMscL in DDM (shaded blue) compared to the modelled distances simulated from the x-ray (closed state) crystal structure (PDB 2OAR) with MtsslWizard (red line).

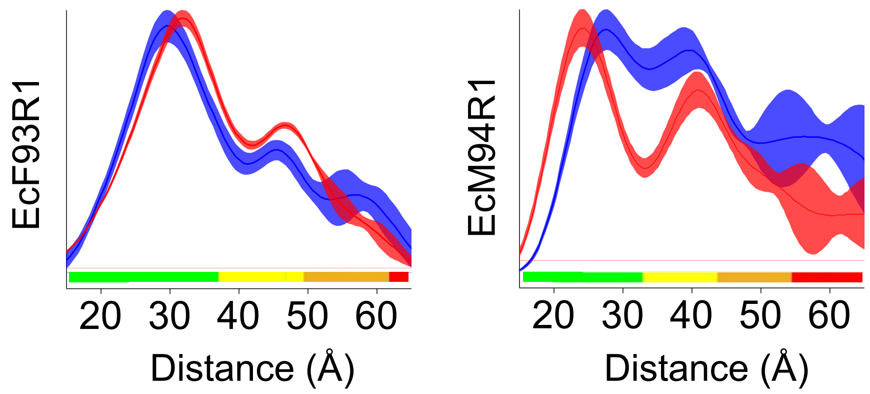

Fig. S3: EcMscL mutants PELDOR distance distribution comparison in DDM detergent (shade blue) and in nanodiscs (NDs) (shaded red).

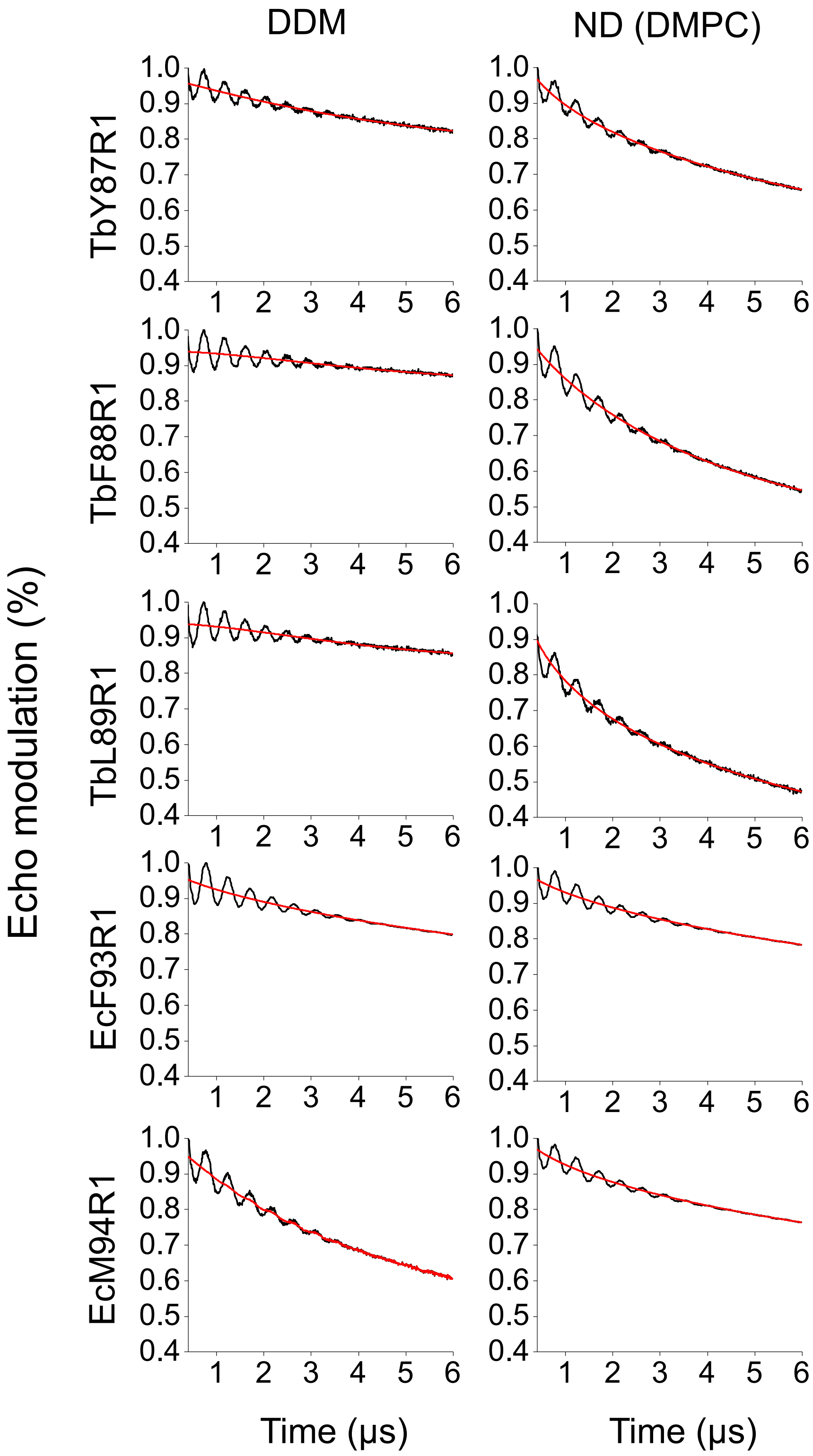

Fig. S4: Raw 3p-ESEEM data (black lines) and stretched exponential background fits (red lines).

		1	2	3	4	5
M. tuberculosis	1		29.45	21.66	29.75	68.99
E. coli	2	39		21.99	47.45	25.31
M. acetivorans	3	62	45		30.77	20.99
S. aureus	4	45	18	39		28.03
M. leprae	5	11	34	69	40	

Table S1: Pairwise alignment comparison between MscL orthologues. % sequence identity is shown in the upper/right half of the table and sequence residue gaps are shown in the lower/left half.

a.

Mutant	D_1; D_2; D_2/D_1 in DDM (Å)	D_1; D_2; D_2/D_1 in NDs (Å)
TbY87R1	29.7; 45.6; 1.55	26.3; 41.1; 1.57
TbF88R1	34.1; 55.8; 1.64	33.6; 53.1; 1.58
TbL89R1	30.0; 48.8; 1.63	27.8; 45.4; 1.63
EcF93R1	29.6; 45.8; 1.55	31.8; 46.9; 1.47
EcM94R1	27.7; 39.1; 1.41	24.1; 40.7; 1.69

b.

D_1 (TbL) – D_1 (EcL)	DDM (Å)	NDs (Å)
TbY87R1 - EcF93R1	0.1	-5.5
TbF88R1 - EcF93R1	4.5	1.8
TbF88R1 - EcM94R1	6.4	9.5
TbL89R1 - EcM94R1	2.3	3.7

Table S2: a. D_1 and D_2 PELDOR distance for each mutant in DDM and in nanodiscs (NDs) and their ratio. The expected ratios for symmetric multimers are 1.41 for a tetramer, 1.62 for a pentamer and 1.73 for a hexamer **b.** D_1 distance differences between distinct mutants for the two orthologues.

Mutant	% change in ^2H accessibility
TbY87R1	(23±5)%
TbF88R1	(28±4)%
TbL89R1	(32±7)%
EcF93R1	-(27±4)%
EcM94R1	-(19±4)%

Table S3: Percentage (%) change in deuterium (solvent) accessibility of MscL in detergent (DDM) following reconstitution in Nanodiscs (DMPC).
