# Supplementary material for: In-Lipid Structure of Pressure-Sensitive Domains Hints Mechanosensitive Channel Functional Diversity
Source: Biophys J. 2020 Jun 23;119(2):448–59. doi: 10.1016/j.bpj.2020.06.012 (PMC7376121; doi:10.1016/j.bpj.2020.06.012)
Supplement: Document S2. Article plus Supporting Material [file mmc2.pdf]

Biophysical Journal
Article

Biophysical Society

In-Lipid Structure of Pressure-Sensitive Domains Hints Mechanosensitive Channel Functional Diversity

Charalampos Kapsalis,[1] Yue Ma,[2,3] Bela E. Bode,[4,*] and Christos Pliotas[1,2,3,*]
[1]Biomedical Sciences Research Complex, School of Biology, University of St Andrews, St Andrews, United Kingdom; [2]Astbury Centre for Structural and Molecular Biology, University of Leeds, Leeds, United Kingdom; [3]School of Biomedical Sciences, Faculty of Biological Sciences, University of Leeds, Leeds, United Kingdom; and [4]Biomedical Sciences Research Complex, School of Chemistry, University of St Andrews, St Andrews, United Kingdom

ABSTRACT The mechanosensitive channel of large conductance (MscL) from *Mycobacterium tuberculosis* has been used as a structural model for rationalizing functional observations in multiple MscL orthologs. Although these orthologs adopt similar structural architectures, they reportedly present significant functional differences. Subtle structural discrepancies on mechanosensitive channel nanopockets are known to affect mechanical gating and may be linked to large variability in tension sensitivity among these membrane channels. Here, we modify the nanopocket regions of MscL from *Escherichia coli* and *M. tuberculosis* and employ PELDOR/DEER distance and 3pESEEM deuterium accessibility measurements to interrogate channel structure within lipids, in which both channels adopt a closed conformation. Significant in-lipid structural differences between the two constructs suggest a more compact *E. coli* MscL at the membrane inner-leaflet, as a consequence of a rotated TM2 helix. Observed differences within lipids could explain *E. coli* MscL's higher tension sensitivity and should be taken into account in extrapolated models used for MscL gating rationalization.

SIGNIFICANCE Despite their reported significant functional differences, two orthologous mechanosensitive channels of large conductance (MscL) originating from two different bacterial species (*Escherichia coli* and *Mycobacterium tuberculosis*) are expected to adopt a very similar structure. Mechanical sensing and response of mechanosensitive channels is highly sensitive to modifications occurring at the entrance of transmembrane nanopockets of these proteins. Here, using pulsed electron-electron double resonance (or double electron-electron resonance) and three-pulse electron spin envelope echo modulation spectroscopy, we found within lipid membranes that substantial structural differences exist within the pressure-sensitive domains of these mechanosensitive channels, which may account for their large discrepancies in their tension sensitivity and therefore functional diversity.

INTRODUCTION

Mechanosensitive (MS) channels form pores in the cell membrane and convert mechanical stimuli into biochemical responses (1,2). Their importance is demonstrated by their ubiquity in all kingdoms of life. In prokaryotic cells, they alter their structure during cell membrane deformations to facilitate equilibration of the osmotic pressure between the opposite sides of the membrane through the flux of solutes (3). In humans, they are involved in hearing, touch, and cardiovascular architecture (4,5). Bacterial MS channels were first reported in the late 1980s (6), and the first MS channel to be cloned was the MS channel of large conductance (MscL) (7), which is found ubiquitously in bacteria and archaea, and is absent from eukaryotes. *Escherichia coli* MscL (EcMscL) consists of a nonselective pore which could reach an estimated diameter of ~30 Å (8) when ~12 mN/m tension is applied, resulting in ~3 nS (7) of conductance. Such a large pore opening in the membrane could allow solutes and even small proteins to pass through (9), which makes MscL an attractive drug target (10) and delivery system (11). In contrast, *Mycobacterium tuberculosis* MscL (TbMscL) requires substantially higher tension to be applied (approximately double) to reach full opening in

Submitted October 30, 2019, and accepted for publication June 10, 2020.
*Correspondence: beb2@st-andrews.ac.uk or c.pliotas@leeds.ac.uk
Editor: Sudha Chakrapani.
https://doi.org/10.1016/j.bpj.2020.06.012
© 2020 Biophysical Society.
This is an open access article under the CC BY license (http://creativecommons.org/licenses/by/4.0/).

either giant unilamellar vesicles or spheroplasts (12–14), despite it presenting similar pore dimensions and total conductance to EcMscL.

To date, two crystal structures of pentameric MscL orthologs in a closed state have been reported, orthologs from *M. tuberculosis* (TbMscL) (Protein Data Bank, PDB: 2OAR) (15) and archaeal *Methanosarcina acetivorans* (PDB ID: 4Y7K) (16), both obtained from detergent purified samples. Although their overall structural architecture is similar, they present significant functional differences (16). The TbMscL channel structure was the first to be solved and served as a structural model for interpreting functional, computational, biophysical, and spectroscopic observations obtained for the majority of other MscL orthologs, mostly EcMscL, for which the TM domain structure remains unknown. EcMscL presents high amino acid sequence similarity (∼46%) with TbMscL (Fig. 1 *a*; Table S1). Hence, in studies in which EcMscL point mutations were included either for functional, biophysical, and/or structural studies, they were rationalized according to this juxtaposition and were projected upon the TbMscL structure (8,17–28), mostly using computationally derived homology models based on sequence alignments. Nevertheless, despite their high sequence similarity, it has become clearly apparent that they display substantially different 1) functional behaviors in vivo, as demonstrated by phenotypic

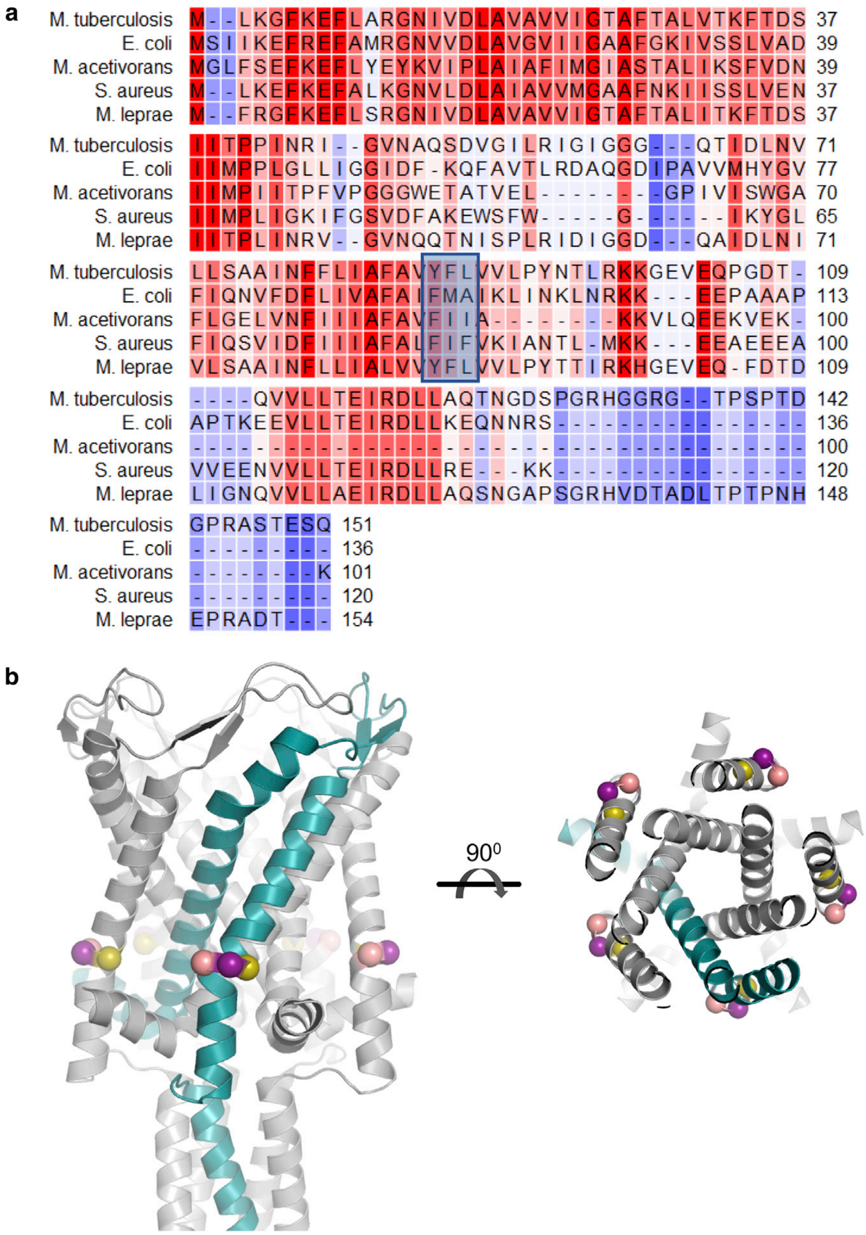

FIGURE 1 Sequence alignment and site selection strategy. (*a*) Protein sequence alignment of MscL orthologs. Conserved residues are highlighted in red. The area of interest is highlighted in the light blue box. (*b*) Crystal structure of the TbMscL pentamer (PDB: 2OAR). Zoomed in image of the area of interest from the side (*left*) and the top (*right*). The protein monomer is shown in cyan, Y87 in olive, F88 in purple, and L89 in salmon. To see this figure in color, go online.

analysis (21) and cell viability in hypoosmotic shocks (14,29) and 2) biophysical/biochemical properties in vitro, as revealed by single-molecule-patch-clamp electrophysiology (12–14,30), W-fluorescence (31), mass spectrometry (32), and continuous wave electron paramagnetic resonance spectroscopy (13,14,19). Furthermore, similar observations are also true for other studied MscL orthologs (13,16,29,33). Such functional differences could be also associated with unique lipid membrane composition between the different organisms (i.e., phosphatidylinositol is present in *M. tuberculosis*, but not *E. coli*) or hydrophobic mismatch due to lipid bilayer thickness differences (20), as MscL's structural integrity, folding, and gating properties are affected by lipids (13,24,30,32,34–36).

Hydrophobic nanopockets (NPs) have been previously identified on the transmembrane (TM) surface of MS channels of small conductance (MscS) and MscL and comprise an integral structural feature of the channel's architecture (14,37,38). These NPs are occupied by lipid chains and play an important role in the MS gating mechanism (14,39–41). As long as they are occupied by annular lipid chains, the channel remains closed, but when chain access to the NPs is disrupted, the channel responds by opening its pore. Similar NPs have also been identified in other eukaryotic ion channels, formed by a motif commonly found in MS channels: a kink between an amphipathic helix that lies at the cytoplasmic-TM interface and a pore lining helix (42–44). The model could be potentially used to explain experimental observations in a variety of eukaryotic channels and receptors that seem to form such NPs (45–50). Interestingly, recent studies on Piezo 1 suggest that when the four conserved lysines known to bind phosphatidylinositol phosphates are deleted (i.e., bind phosphatidylinositol phosphates can no longer bind to proximal to Piezo pore NPs), a major loss of inactivation is observed (49). Furthermore, in another study, it was suggested that upon tension application to Piezo within lipid bilayers, phosphatidylethanolamine lipids located at similar sites, move away from these pockets and outwards in respect to the channel pore (48), in agreement with our model for MscS (39) and MscL (14,43). The first direct experimental evidence of the hypothesis that lipid chain disruption from accessing the NPs leads to a structural channel response, known as the "lipid moves first" model (39), has been obtained for the TbMscL channel (14), the ion channel with the highest pressure activation threshold known in nature. When the entrance of the MS channel's NPs, which are located distal to the channel pore, is disrupted either by covalent cysteine modification or mutagenesis, the channel senses this NP lipid discrepancy and responds by releasing the protein's stored elastic energy, gained from its prior bilayer compression, and opening its pore (expanded and subconducting state) (14,39,43). The "lipid moves first" model expands on the "force-from-lipid" model (51,52) and is aligned with the "Jack-in-a-box" model proposed for MscS (53), but provides a molecular description on how specific lipids act on MS pressure-sensitive domains and drive mechanical sensing and response. Therefore, these NP lipids act as pressure safety pins for the channel's stored elastic energy and must move first for mechanotransduction to occur. This mechanical response could be achieved by sterically excluding lipid chains from the NPs with a sulfhydryl side chain (14). Interestingly, this effect could be reversed upon reconstitution into lipid bilayers (lateral bilayer compression), where lipid chains are forced back to the occupy NPs, whereas removal of the modification restores channel function to wild-type (WT) levels (14). The effect was observed for one site (out of the total 13 tested and spanning all channel domains), suggesting the importance of applying localized pressure to MscL to promote global structural and functional channel responses. Recently, a CryoEM study of MscS showed that the lipid bilayer is shifted by ∼14 Å, and it was suggested that bulk membrane lipids could directly access and block the channel's pore (54). This model neither includes the NPs within the channel's TM region, nor does it explain the presence of resolved lipid chains residing within MscS' NPs found in cryo-electron microscopy (CryoEM) and x-ray structures of multiple MscS states solved by several independent groups (39,40,55–58). A similar shift of the lipid bilayer for MscS to the one observed in the CryoEM model (54) was first reported in an MD simulation study in which the mid-plane bilayer position relative to MscS stabilized at a level ∼10 Å above the position chosen in previous simulations (59). A similar bilayer shift for both the closed and open MscS states was demonstrated in a study that combined structural, computational, and functional observations to propose a gating model for MscS (39). In particular, few inner-leaflet lipids were shown to partly disengage from the residual cytoplasmic-facing bulk lipid bilayer to access and intercalate into the NPs, thereby gating MscS (39). This membrane shift toward the periplasmic side was also evident from the first CryoEM MscS structure solved in lipid nanodisks (40). There, the membrane was also shifted toward the periplasmic side by >10 Å, and two and one lipids per subunit were resolved in the NPs and the anchor domain, respectively. Inner-leaflet lipids accessing the NPs even when they are located >10 Å away may also be facilitated by increased local curvature, observed in the MD simulations (39), but not in the CryoEM model, in which the bilayer is flat (54). This curvature shift may not have the same effect on MscL though, where the amphipathic helix responsible for structurally forming the NPs, and thus important for MscL's mechanosensitivity, lies within or at the interface of the lipid bilayer (28).

Pulsed electron-electron double resonance (PELDOR) or double electron-electron resonance (DEER) spectroscopy has been proven to be a powerful method for investigating protein structure and dynamics (60–63). In particular, the method has been successful in assigning conformation,

oligomerization, and folding under physiological conditions or within lipid environment for a variety of membrane proteins (14,37,38,64–72) and is capable of offering subangstrom accuracy (39,60). Electron spin envelope echo modulation (ESEEM) spectroscopy has been used for measuring site-specific deuterium (solvent) accessibility in membrane proteins (14,73–75), and the method is a valuable tool for identifying lipid/detergent buried or exposed membrane protein sites.

We hypothesized that functional differences between MscL orthologs should be associated with structural differences and localized proximate to the NPs. We here reconstitute EcMscL and TbMscL in lipid nanodisks (NDs) and employ PELDOR and three-pulse ESEEM (3pESEEM) spectroscopy to obtain structural information from their TM domain and compare their NP region in in-lipid closed state. We observe significant structural discrepancies at the entrance of the NP (allosteric site), which should account for the substantial functional diversity among MscL orthologs.

MATERIALS AND METHODS

n-Dodecyl-β-D-maltopyranoside (DDM) anagrade was obtained from Anatrace (Maumee, OH) or Glycon (Luckenwalde, Germany). Isopropyl-β-D-thiogalactoside was obtained from Formedium (Norfolk, UK), and tris(2-carboxyethyl)phosphine (TCEP) was obtained from Thermo Fisher Scientific (Waltham, MA). The S-(2,2,5,5-tetramethyl-2,5-dihydro-1H-pyrrol-3-yl)methyl methanesulfonothioate (MTSSL) spin label was obtained from Toronto Research Chemicals (North York, Canada), dimyristoyl phosphatidylcholine (DMPC) (14:0/14:0) was purchased from Avanti Polar Lipids (Alabaster, AL), and Biobeads were purchased from Bio-Rad Laboratories (Watford, UK). All other chemicals were obtained from Sigma-Aldrich (Dorset, UK) unless otherwise stated.

Site-directed mutagenesis and protein expression

All mutants were generated using a modification of the Stratagene QuikChange protocol, as described previously (76). Both TbMscL and EcMscL were carrying a C-terminal 6 $\times$ His-tag. TbMscL mutants were generated on a pJ411:140126 vector, whereas the EcMscL mutants were generated on a pET-52b vector. Subsequently, *E. coli* BL21 (DE3) cells (Thermo Fisher Scientific, Oxford, UK) were transformed with the modified plasmids and the cells were grown in 0.5 L of Luria-Bertani (LB) broth medium at 37°C until they reached an OD600 $\approx$ 0.8–0.9. Protein expression was then induced by the addition of 1 mM isopropyl-β-D-thiogalactoside for 4 h at 25°C. Finally, the cells were harvested by centrifugation at 4000 $\times$ *g*, and the pellets were stored at -80°C.

Purification and spin labeling

Protein purification was performed as described previously (14,38,39,60). Briefly, the cell pellets were thawed, resuspended in phosphate-buffered saline, and lysed with a cell disrupter at 30 Kpsi. The resulting suspension was centrifuged at 4000 $\times$ *g* for 20 min and, afterwards, the supernatant was centrifuged again for 1 h at 100,000 $\times$ *g*. The resulting membrane pellet was resuspended and mechanically solubilized in solubilization buffer (50 mM sodium phosphate (pH 7.5), 300 mM NaCl, 10% v/v glycerol, 50 mM imidazole, and 1.5% w/v DDM) and then left for 1 h at 4°C with gentle shaking. The solution was subsequently centrifuged at 4000 $\times$ *g* for 20 min, and the resulting supernatant was passed through a Ni^{2+}-nitrilotriacetic acid column containing $\sim$0.75 mL Ni^{2+}- NTA beads. Afterwards, the column was washed with 10 mL of wash buffer (50 mM sodium phosphate (pH 7.5), 300 mM NaCl, 10% v/v glycerol, and 0.05% w/v DDM) and then with 5 mL of wash buffer with 3 mM TCEP was added to it. Then, 3 mL of wash buffer supplemented with MTSSL at a 10$\times$ excess of the expected protein concentration was added to the column and was left to incubate at 4°C for 2 h. Subsequently, the protein was eluted with wash buffer containing 300 mM imidazole before being subjected to size exclusion chromatography (SEC) using a Superdex 200 column (GE Healthcare, Chicago, IL) and a SEC buffer (50 mM sodium phosphate (pH 7.5), 300 mM NaCl, and 0.05% w/v DDM). Finally, the pure protein collected from the SEC column was concentrated to a monomer concentration of $\sim$800 μM. Spin labeling efficiency of the proteins was quantified with a method previously described (77). To be used for the PELDOR measurements, samples were diluted 1:1 with deuterated ethylene glycol, and 70 μL of the mixture wewasre loaded in 3 mm (OD) quartz tubes and flash frozen in liquid N_2.

Reconstitution in nanodisks

Nanodisk reconstitution was performed as described previously (14,37,60). Briefly, purified and spin-labeled protein cysteine mutants were mixed with the membrane scaffold protein MSP1D1 (78) and presolubilized DMPC lipids in a 1:2:160 respective molar ratio. The mixture was diluted to 3–4 mL with lipid buffer (50 mM sodium phosphate (pH 7.5), 300 mM NaCl, 1% Triton X-100) and incubated at 25°C for 30 min. Then, 0.8–1 g of Biobeads (Bio-Rad Laboratories) were added per 1 mL of the mixture and left incubating with light agitation at 25°C for 4 h. Finally, Biobeads were removed and concentrated to a final volume of 35 μL. For PELDOR measurements, samples were diluted 1:1 with deuterated ethylene glycol, and 70 μL of the mixture was loaded in 3 mm (OD) quartz tubes and flash frozen in liquid N_2.

Distance modeling

Spin labeling and distance modeling in silico was performed on the TbMscL crystal structure (2OAR) using the MtsslWizard plugin in PyMOL (79). Residues were first mutated to cysteines and subsequently spin labeled using the “thorough search” option for the MTSSL rotamers while Van der Waals restraints were set to tight.

PELDOR measurements and data analysis

All measurements were performed using a Bruker ELEXSYS E580-pulsed Q band (at 34 GHz) spectrometer at 50 K with a TE012 cavity. The two frequencies used, detection (ν_A) and pump (ν_B), had an offset of 80 MHz between them and the pulse sequence was $(\pi/2)_A$ - τ_1 – π_A - (τ_1 + t) - π_B - (τ_2 – t) - π_A - τ_2 - echo (80) with a shot repetition time of 3 ms. Detection pulse lengths were set to 16 and 32 ns for $(\pi/2)_A$ and π_A, respectively; τ_1 was 380 ns; and the π_B pulse length was set to either 12 or 14 ns. The pump frequency was set to coincide with the maximum of the nitroxide spectrum and the resonance frequency of the resonator. The detection frequency was set to an 80 MHz lower frequency. Data were subsequently analyzed using the MATLAB (The MathWorks, Natick, MA) plugin DeerAnalysis 2016 (81) by Tikhonov regularization (82) once the raw time domain data were background corrected. Furthermore, we employed the DeerAnalysis validation tool as described previously (70). We varied the background-fitting starting point from 5 to 80% of the length of the time domain trace in 16 steps while adding 50% random noise at 50 trials per fitting step, which resulted in 800 trials per trace in total. Finally, we discarded data sets that were more than 15% above the lowest, and thus best, root mean-square deviation (RMSD) value. Some background-fitting functions yielded the best RMSD for an unphysical, continuously rising background corresponding to negative

concentration. This was associated with real but incomplete dipolar modulation of the trace rather than artifacts. Relaxation is limiting the scope to extend the time trace and cutting portion of the trace that displays the incomplete oscillation would greatly compromise the resolution of longer distances in the resulting distance distributions. Therefore, for all data sets, the best fit for the full traces of was consistently used. This did not lead to significant changes to the distance distribution with respect to fitting the background to the latter two-thirds of the trace (14).

Multispin effects that can hamper data analysis in samples with more than two spins per protein complex (60,83) can be suppressed by power scaling (84), reduced excitation bandwidth (85), or sparse labeling (70). For pentamers and the excitation bandwidth achieved here, power scaling proved sufficient to produce distance distributions with minimal multispin artifacts.

ESEEM measurements and data analysis

Sample preparation is identical to PELDOR measurements. All measurements were performed using a Bruker ELEXSYS E580 X band (9.5 GHz) spectrometer at 80 K with a 3-mm split ring (MS3) or 4-mm dielectric (MD4) resonator. The 3pESEEM was recorded at the maximum of the nitroxide spectrum with pulse sequence $\pi/2$-τ- $\pi/2$-T- $\pi/2$- τ-echo. Each $\pi/2$ pulse was set to 16 ns, interpulse delay τ was set to $\sim$140 ns (two times the reciprocal ^{1}H Larmor frequency at the magnetic field used), corresponding to a blind spot of the proton ESEEM, and delay time T was incremented in 12 ns steps. The obtained time domain signal traces were background corrected with a stretched exponential decay by first subtracting the decay function and then dividing by it to retain the modulation depth information. Despite slight imperfections in the corrected traces, this proved more robust than the use of polynomial background functions. These traces were apodized with a hamming window function and zero filled before being Fourier transformed (86). Relative deuterium accessibility was then taken from the absolute of the spectrum at the deuterium Larmor frequency at the magnetic field the trace was recorded. The errors were estimated from the RMSD of the imaginary of the phase-corrected and -normalized traces.

RESULTS

We first generated two (F93C and M94C) EcMscL cysteine mutants and compared with equivalent (by sequence alignment) TbMscL variants. Sequence alignments of previous studies between EcMscL and TbMscL showed that EcM94 aligns with TbF88 (Fig. 1 *a*; (12,15,87)). Y87, F88, and L89 mutants are in the proximity or entrance of TbMscL's NPs (Fig. 1 *b*), with modifications of the latter known to have significant allosteric effect on TbMscL gating (14). Because EcM94 aligned with TbF88, we selected this *E. coli* mutant along with F93 to "structurally" align TbMscL with EcMscL at the NP equivalent region dictated by sequence alignment performed among MscL orthologs (Fig. 1 *a*). We further reconstituted MscL channels in DMPC NDs to ensure that both orthologous channel proteins adopt a closed conformation, consistent with all 13 TbMscL ND-reconstituted variants (presence of bilayer compression) assessed by PELDOR and found to be in a closed conformation and pentameric state (14). In the same study, the use of *E. coli* polar lipid extract containing $\sim$67% phosphatidylethanolamine, 23.2% phosphatidylglycerol, and 9.8% of cardiolipin lipids of variable acyl chain lengths and saturation degrees resulted in the same closed TbMscL state as revealed by PELDOR, which retrieves the distance dependent dipolar coupling between protein sites, thus providing a direct measure of channel conformation (Fig. S1; (14)). Previously, continuous wave electron paramagnetic resonance accessibility measurements, which report on the local environment of the label, suggested that in C14 lipids, EcMscL adopts an intermediate closed conformation compared to C18 lipids (20). Therefore, these two studies first hinted differences in structural behavior between the two MscL orthologs.

In detergent solution, spin-labeled EcMscL mutants F93R1 and M94R1, with R1 denoting spin-labeled cysteine modification thereafter, displayed very little oscillation despite their large modulation depths (Fig. 2), which translates into broader and less defined distance distributions (Fig. 3; (70)). The three TbMscL (Y87R1, F88R1, and L89R1)-equivalent mutants in detergent solution displayed deep modulations and visible oscillations in the raw time trace (Fig. 2), which lead to clearly defined and reliable distance distributions (Fig. 3).

The distance distributions of TbY87R1 and TbF88R1 along with 10 other modified location variants agree well with in silico model distances on the pentameric TbMscL-closed conformation x-ray crystal structure with the exception of L89R1, which adopted an expanded state in detergent solution (Fig. S2; (14)). The distance distribution of EcF93R1 is broad, but it coincides more with that of TbY87R1 while also displaying a probability of long distances (Figs. 2 and 3; Table S2). WT EcMscL carrying a His-tag on its C-terminus and solubilized in DDM, similar to our construct and sample, was in a monodisperse state consistent with a pentamer, as shown by mass spectrometry (32). In the case of EcM94R1, the lack of clear oscillations in the raw data gives rise to a very broad distance distribution (70). Nevertheless, those peaks seem to agree better with the distance distribution of TbL89R1 rather than that of TbF88R1, and our PELDOR data showed a significant discrepancy between these two variants (Fig. 3; Table S2). Except for EcM94R1, in which the broad distribution hampers the extraction of exact distances, the ratio between the two distance peaks agrees well with the value of 1.62 expected for a symmetric pentamer (Fig. 3; Table S2).

Upon reconstitution into lipid NDs, both modified EcMscL variants surprisingly were more structurally defined, as seen by their time domain signals (Fig. 2 *b*). The lack of clear oscillations in EcF93R1 further results in a broad distance distribution, which better agrees with TbF88R1 (Fig. 4; Table S2). TbMscL mutants are displaying time traces similar to the ones in detergent solution (Fig. 2), and the distance distributions remain well defined in NDs (Fig. 4; Table S2; (14)). EcM94R1 reconstitution in NDs gives rise to a slightly clearer oscillation than in DDM, which results in a more defined distance distribution, suggesting the channel adopts a closed conformation, more in agreement with TbL89R1 rather than TbF88R1 (Figs. 2 *b*

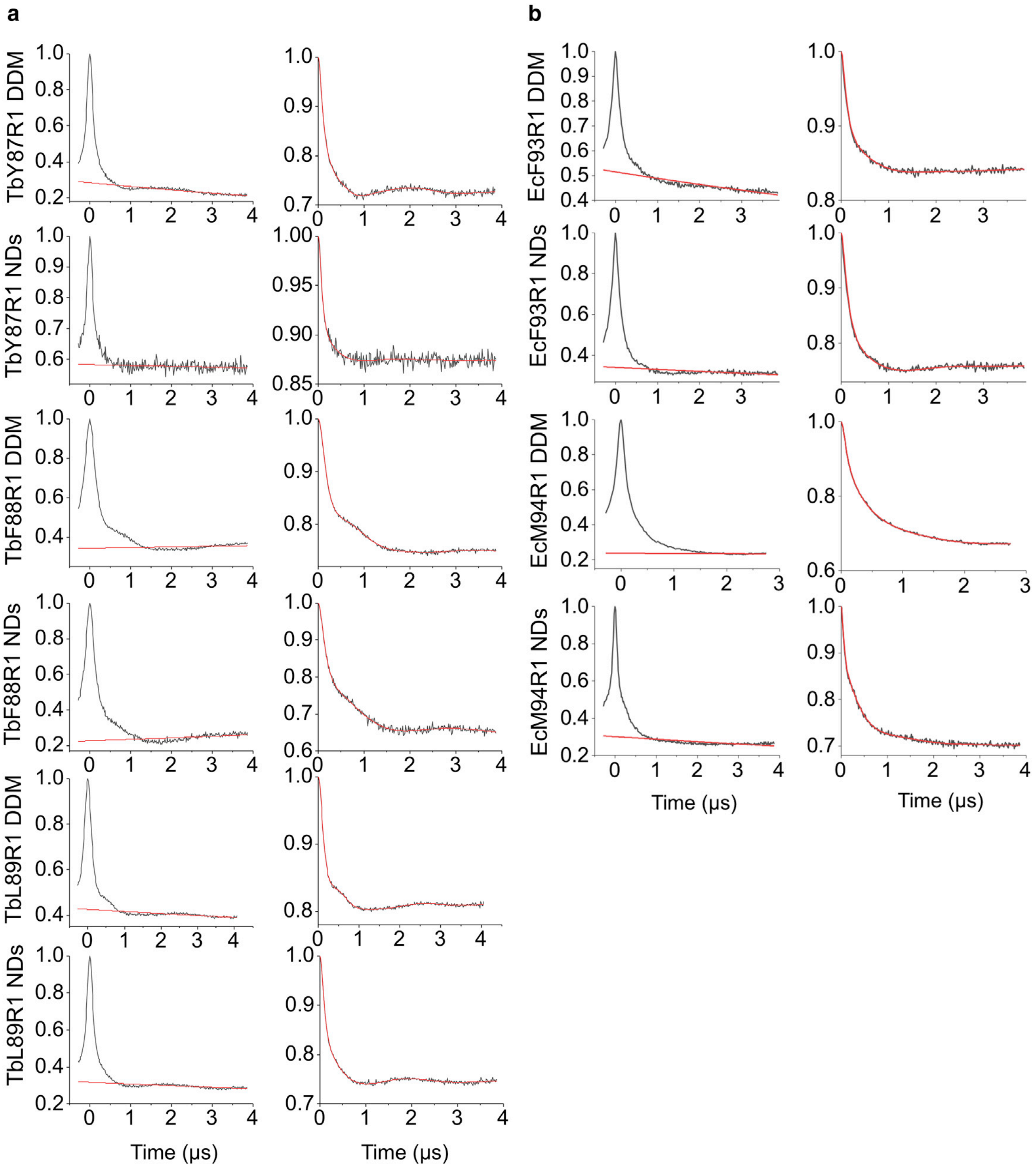

FIGURE 2 Raw and background corrected PELDOR time domain data for TbMscL and EcMscL variants. (*a*) TbMscL-raw PELDOR time domain data (14) with the background in red from left (*first column*) and corrected traces with the fitting function in red (*second column*). (*b*) . EcMscL mutants' raw PELDOR time domain spectra with the background in red (*third column*) and corrected traces with the fitting function in red (*fourth column*). To see this figure in color, go online.

and 4; Table S2). Except for EcM94R1 showing significant contributions of long distances, the ratio between the two expected distance peaks agrees well with the value of 1.62 expected for a symmetric pentamer (Fig. 3; Table S3). We refrain from overinterpreting the distance distribution of EcM94R1 that might arise from a number of distinct conformers or a very broad distribution of conformers in DDM with a reduced number of conformers or a reduced flexibility in lipid environment. Nevertheless, if the D_1 peak is around 2.5 nm, D_2 would be expected around 4 nm, and if D_1 was 4 nm, D_2 should be around 6.5 nm. We can indeed not exclude two conformations of EcM94R1 being present with D_1 values of 2.5 and 4.0 nm but do not have the resolution to unequivocally prove the distance probability around 6.5 nm is significant.

To investigate the solvent exposure of the labeled sites, we performed 3pESEEM measurements. The buffer contains 50% deuterated ethylene glycol; thus, R1 exposure to deuterium is assessed by the intensity of the modulation of the electron paramagnetic resonance signal caused by

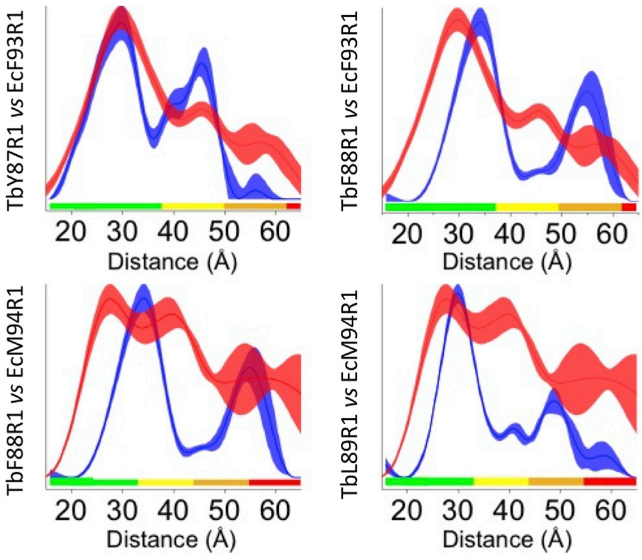

FIGURE 3 Comparison of PELDOR distance distributions in DDM solution. TbMscL (*blue*) versus EcMscL (*red*). Shaded areas correspond to mean ± 2σ confidence intervals of the measured distributions (calculated using the DeerAnalysis validation tool), and color bars indicate the reliability of the measured distance ranges (calculated using DeerAnalysis), depending on the measurement time windows. In each comparison figure, the color bars correspond to the measurement with the shortest time window, i.e., lowest confidence. To see this figure in color, go online.

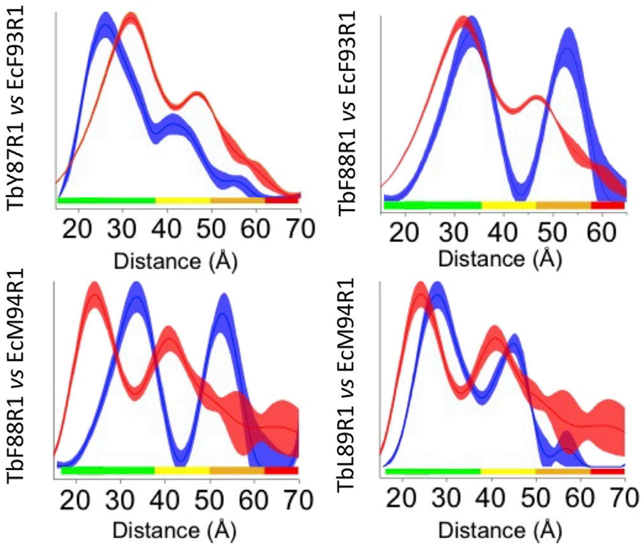

FIGURE 4 Comparison of PELDOR distance distributions in DMPC NDs. TbMscL (*blue line*) versus EcMscL (*red line*) PELDOR distance distributions in MSP1D1 DMPC NDs. Shaded areas correspond to mean ± 2σ confidence intervals of the measured distributions (calculated by the DeerAnalysis validation tool), and color bars indicate the reliability of the measured distance ranges (calculated by DeerAnalysis), depending on the measurement time windows. In each comparison figure, the color bars correspond to the measurement with the shortest time window, i.e., lower confidence. To see this figure in color, go online.

nearby ($\leq$1 nm) deuterium nuclei. After reconstitution into NDs, deuterium accessibility was increased for TbMscL mutants (Figs. 5, *a* and *c* and S4) and reduced for EcMscL mutants (Fig. 5 *b*; Table S3). The increases in deuterium accessibility were 23, 28, and 32% for TbY87R1, TbF88R1, and TbL89R1, respectively, whereas the decreases were 27% for EcF93R1 and 19% for EcM94R1 (Figs. 5, *b–d* and S4; Table S3).

DISCUSSION

Force in the membrane is mediated through the lipids; a well-defined transmembrane (TM) region seems to play a major role in the regulation of mechanically gated channels, which share the common mechanism of mechanosensitivity (14,39,43). These highly pressure-sensitive domains or NPs appear to be an integral part of MS channels of similar (orthologs) (15,16) or distinct to MscL's structural architecture pro- and eukaryotic ion channels (37–40,42,44). Because of the high pressure sensitivity of the NPs capable of inducing structural responses, even subtle differences within the allosteric NP site could lead to significant functional differences in MS channels. Here, we compared the NPs of the two most extensively studied MscL orthologs (i.e., TbMscL and EcMscL) and use PELDOR to structurally align them. These proteins present significant differences in their function and biophysical properties in vitro and in vivo. Such differences may originate from lipid composition membrane variability between the two species.

Computational models using different orthologs to rationalize lipid-protein interactions involved in channel gating are a useful tool; however, they may not capture all subtleties of the interactions that underpin gating, especially when they are based on homology-derived protein models. Importantly, in the case of highly pressure-sensitive NPs, subtle differences may be so small that they approach the scale of computational artifacts. In this case, the derived models may either not provide an accurate representation of the molecular detail of interactions involved in gating or unreliable assumptions may be made on the basis that subtle differences proximate to the allosteric site are negligible for channel gating. Invalid assumptions could be even further amplified by artifacts induced by ambiguous sequence alignments or structure registers in model building. Consequently, nonoptimal orientation of residues exposed or accessible to the lipid bilayer and thus crucial for gating could influence data interpretation and scientific conclusions.

Major functional differences and lipid membrane requirements have been reported between EcMscL and TbMscL orthologs despite an expected similar architecture (12,13,30). Here, given their substantial functional differences, e.g., in pressure sensitivity reported by multiple groups, we hypothesized these should be linked to structural differences (subtle or major), proximate to their NP allosteric site. We therefore employed PELDOR to assess their structure within that region and 3pESEEM to measure and compare

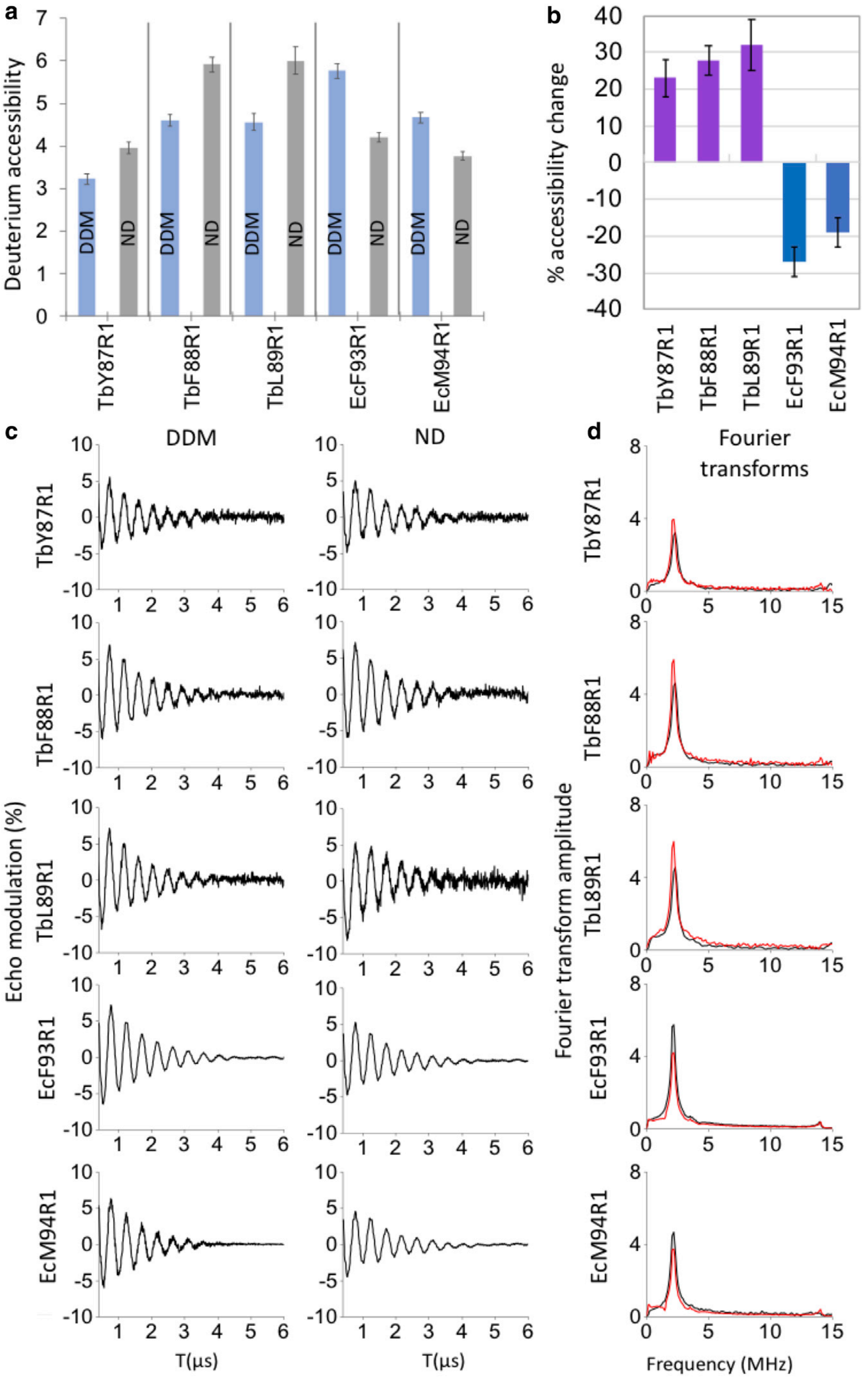

FIGURE 5 Comparison of 3p-ESEEM ^{2}H (or solvent) accessibility of MscL orthologs in detergent and lipid NDs. (*a*) ^{2}H accessibility of Tb and EcMscL sites in DDM (*light blue*) and NDs (*gray*) columns calculated as the peak intensity of ^{2}H in the frequency domain spectra. (*b*) Percent change of ^{2}H accessibility after reconstitution of the detergent (DDM) MscL samples into NDs (DMPC). (*c*) Background-corrected ESEEM time domain traces. (*d*) Fourier transform frequency spectra. Detergent (*black line*) and ND (*red line*) samples. The peak at 2.6 MHz corresponds to ^{2}H Larmor frequency. Errors in (*a*) and (*b*) are calculated based on the background noise of the time domain trace after a fitting with a stretched exponential decay function. To see this figure in color, go online.

the solvent accessibility of key residues. We further performed these measurements within lipid bilayers (i.e., NDs) because 1) it is a biologically relevant environment for TM domains of membrane channels such as MscL and 2) both channels adopt a similar conformational state (closed). This allowed us to perform a direct valid comparison between the two channels and investigate the existence of any structural differences between their NP-forming regions, for which only a TbMscL full-length x-ray model is available (15) in contrast to the unknown TM domain of EcMscL. Lipids play a crucial role in mechanosensation as the carriers of tension transmission, and as such, the detailed description of their molecular interaction with pressure-sensitive MS channel regions is of crucial importance. PELDOR offers the advantage of providing vital structural information for proteins for which a structural model is not available from either by x-ray crystallography and/or CryoEM. Here, we obtained structural information about

important residues around the pressure-sensitive NP region (allosteric site) facilitating 3pESEEM to measure solvent accessibility and also gain insights into their relative orientation in respect to the lipid bilayer. The latter, along with obtained PELDOR distance measurements for these residues, allowed a structural alignment of these two orthologous MscL channels within a crucial for gating, and thus functional, region.

Amino acid sequence alignments paired EcM94R1 with TbF88R1 (8,17–25,28). According to our PELDOR data, the distance distributions of EcM94R1 structurally align with those of TbL89R1 rather than TbF88R1 in both detergent and ND (closed-state) samples. Our finding is in contrast with previous assumptions that EcM94 is equivalent to TbF88 and suggests that there is an ~90° degree deviation between the two residues. The latter is also supported by 3pESEEM measurements, which suggest that all TbMscL residues (i.e., Y87, F88, and L89) (Fig. 1 *b*) become more solvent exposed (or deprotected), whereas in contrast, both EcMscL residues (i.e., F93 and M94) become more buried (or protected), when both channels transit toward a closed conformation in NDs (Fig. 5 *b*; Table S3). This is consistent with a substantial inner-leaflet TM2-helical rotation for both channels in agreement with previous suggestion for TbMscL (14). However, comparison of the subsequent residue 3pESEEM accessibilities for EcMscL and TMscL strongly suggests a substantial difference in relative orientation of the structurally equivalent TM2 residues within their in-lipid closed-state structures (Fig. 5 *b*). Consequently, this should have implications for the analysis and conclusions of previous studies which based their model assumptions for this residue similarity between TbMscL and EcMscL.

Although the oscillations of EcM94R1 signal amplitude in the DDM sample are dampened, leading to a broad distance distributions and suggesting multiple states in detergent (70), PELDOR reveals this residue is closer "structurally aligned" with TbL89R1 than TbF88R1. We previously demonstrated (14) that TbL89R1 adopts an expanded conformation in detergent that is reversed upon reconstitution in lipid bilayers (NDs or liposomes) (14). Both EcM94R1 and TbL89R1 adopt a closed conformation within ND reconstituted samples, and PELDOR distances are consistent with TbL89R1 rather than with TbF88R1, presenting a 6- and 12-Å difference in their modal distances, respectively.

"Structural" alignment of TbMscL L89 with EcMscL M94 would suggest that M94 is also located at the entrance of the EcMscL's NPs, and therefore, EcM94R1 modification may equally affect channel's gating behavior and pressure threshold similar to TbL89R1 (14). This could explain the similar behavior observed in the distance distributions of EcM94R1 upon lipid reconstitution. Evidently, ND-reconstituted EcM94R1, unlike DDM, adopts a more defined conformation (Figs. 4 and S3). In contrast, EcF93R1, located next to EcM94, presents no significant structural differences between DDM and DMPC NDs, suggesting the protein adopts the same conformation (closed) in both environments. The EcF93R1 modification does not therefore affect the channel state, unlike the adjacent EcM94R1 modification, which seems to have an effect on EcMscL structure in DDM (Fig. S3). This suggests that the observed structural change is site specific and only due to EcM94C modification. PELDOR revealed that EcM94 is structurally equivalent to TbL89 in lipids (Figs. 2 and 4), which, when modified with MTS or mutated to a bulky tryptophan, has shown to cause dramatic effects on TbMscL structure and function, whereas when adjacent, TbMscL residues are modified have no effect on structure (14). It is important to note that these observed distance changes for both constructs and the equivalent residues (EcM94 and TbL89) are much larger and way beyond the length of the MTSSL spin label, excluding that these changes in PELDOR distance distributions could have been caused by MTSSL's rotameric flexibility or restrictions in the presence of lipids and/or detergent.

The larger distance shortening observed for EcM94R1 when compared to TbL89R1 could be associated with the differences in the functional behavior between the two orthologs and, in particular, that WT EcMscL opens at significantly lower pressure activation thresholds (~50%) than WT TbMscL (12,13). This suggests that the former is more sensitive to tension changes and requires significantly smaller forces to induce gating transitions. Hence, upon reconstitution in lipid bilayers, EcMscL has greater conformational (expansion or contraction) space than TbMscL, which could result in the larger distance reduction for EcM94R1 when lateral bilayer compression is induced by the surrounding bilayer within NDs, which is in agreement with our PELDOR observations (Figs. 3, 4, and S3; (14)). EcM94R1 in DDM adopts multiple conformational states, without excluding the possibility though, of multiple oligomeric states. However, when the channel was reconstituted in lipids adopted a defined state with substantially shorter distances and a D_2/D_1 distance ratio of 1.69 (Table S2 a), that is consistent with a pentamer or hexamer, in agreement with a previous pentameric EcMscL in DDM observed by native mass spectrometry, for a similar EcMscL C-GFP construct (32). Therefore, possibly a single oligomeric state (pentamer) and multiple conformations exist within the DDM solution, whereas a defined structural state is forced by the presence of bilayer compression (closed) rather than multiple oligomeric states altered because of the presence of lipids (Fig. S3). Interestingly, modification of EcM94C, or surrounding residues, with different MTS modifiers did not seem to have an effect on the cell viability of EcMscL (26,27). However, the molecular mechanism by which these inner-membrane protein cysteines are modified (or labeled) in vivo in Gram-negative bacteria, because MTS is expected to get immediately reduced upon entry to

the periplasm, is unclear (88,89). 3p-ESEEM deuterium accessibility measurements are further supportive of PELDOR findings and provide further insights on the structure and solvent accessibility of the important to gating NP channel region. In particular, three subsequent residues that form a single helix in TbMscL, e.g., TbY87, TbF88, and TbL89, become more deprotected (or solvent exposed) when reconstituted into NDs compared to DDM. Interestingly, PELDOR revealed that the structurally equivalent EcMscL residues F93 and M94 both display an inverse effect to TbMscL structurally equivalent residues by becoming substantially more protected and presenting lower deuterium (solvent) accessibility in lipids (Fig. 5 *b*; Table S3). In case this was a site-specific effect, then we should observe variability among these adjacent residues. However, that was not the case, as for all tested EcMscL and TbMscL residues, a similar accessibility pattern was observed, suggesting the NP region is influenced by the presence of lipids in both channels, though through completely contrasted ways. The latter is surprising for equivalent residues and domains of two very closely sequence-related and structurally similar orthologous ion channels, but they reportedly present substantial functional differences.

Amino acid sequence alignment and derivation of computational models as initial approaches based on orthologous experimental structures are very useful tools in understanding protein-function-structure relationships. Our PELDOR and ESEEM data suggest that this information should be treated with caution because these methods rely on sequence alignment not equivalent to their registry or structural alignment. PELDOR could offer such structural alignment through direct comparison of different constructs and also within the lipid or native environment in the case of membrane proteins. Furthermore, when these measurements are supported by ESEEM solvent accessibility measurements for individual residues located in force-sensitive NP regions known to be critical for MS function, initial structural models and rationalization/design could be drastically improved, leading to more reliable data analysis and interpretation.

The research data underpinning this publication can be accessed at https://doi.org/10.17630/dbad68d7-7656-4e81-b790-6eb98bdc4f11.

SUPPORTING MATERIAL

Supporting Material can be found online at https://doi.org/10.1016/j.bpj.2020.06.012.

AUTHOR CONTRIBUTIONS

C.P. conceived and designed the project. C.P. and B.E.B., with input from C.K., analyzed and interpreted the data. Experiments were performed by C.K., Y.M., B.E.B., and C.P. C.P. wrote the article with input from B.E.B and C.K.

ACKNOWLEDGMENTS

This project was supported by a Biotechnology and Biological Sciences Research Council grant (BB/S018069/1) to C.P., who was supported by the Royal Society of Edinburgh, Tenovus (T15/41) and Carnegie Trust (OS000256) at the initial stages of this project. Furthermore, C.P. acknowledges support from the University of St Andrews for the C.K. studentship and the University of Leeds and the Chinese Scholarship Council for the Y.M. studentship. B.E.B. and C.P. acknowledge support by the Leverhulme Trust (RPG-2018–397). This work was also supported by previous Wellcome Trust [099149/Z/12/Z] and Biotechnology and Biological Sciences Research Council equipment grants (BB/R013780/1).

REFERENCES

1. Levina, N., S. Tötemeyer, …, I. R. Booth. 1999. Protection of *Escherichia coli* cells against extreme turgor by activation of MscS and MscL mechanosensitive channels: identification of genes required for MscS activity. *EMBO J.* 18:1730–1737.
2. Wilson, M. E., G. Maksaev, and E. S. Haswell. 2013. MscS-like mechanosensitive channels in plants and microbes. *Biochemistry.* 52:5708–5722.
3. Booth, I. R., M. D. Edwards, …, S. Miller. 2007. Mechanosensitive channels in bacteria: signs of closure? *Nat. Rev. Microbiol.* 5:431–440.
4. Li, J., B. Hou, …, D. J. Beech. 2014. Piezo1 integration of vascular architecture with physiological force. *Nature.* 515:279–282.
5. Coste, B., J. Mathur, …, A. Patapoutian. 2010. Piezo1 and Piezo2 are essential components of distinct mechanically activated cation channels. *Science.* 330:55–60.
6. Martinac, B., M. Buechner, …, C. Kung. 1987. Pressure-sensitive ion channel in *Escherichia coli*. *Proc. Natl. Acad. Sci. USA.* 84:2297–2301.
7. Sukharev, S. I., P. Blount, …, C. Kung. 1994. A large-conductance mechanosensitive channel in *E. coli* encoded by mscL alone. *Nature.* 368:265–268.
8. Wang, Y., Y. Liu, …, P. R. Selvin. 2014. Single molecule FRET reveals pore size and opening mechanism of a mechano-sensitive ion channel. *eLife.* 3:e01834.
9. Doerner, J. F., S. Febvay, and D. E. Clapham. 2012. Controlled delivery of bioactive molecules into live cells using the bacterial mechanosensitive channel MscL. *Nat. Commun.* 3:990.
10. Wray, R., I. Iscla, …, P. Blount. 2016. Dihydrostreptomycin directly binds to, modulates, and passes through the MscL channel pore. *PLoS Biol.* 14:e1002473.
11. Iscla, I., C. Eaton, …, P. Blount. 2013. Improving the design of a MscL-based triggered nanovalve. *Biosensors (Basel).* 3:171–184.
12. Herrera, N., G. Maksaev, …, D. C. Rees. 2018. Elucidating a role for the cytoplasmic domain in the *Mycobacterium tuberculosis* mechanosensitive channel of large conductance. *Sci. Rep.* 8:14566.
13. Mukherjee, N., M. D. Jose, …, A. Koçer. 2014. The activation mode of the mechanosensitive ion channel, MscL, by lysophosphatidylcholine differs from tension-induced gating. *FASEB J.* 28:4292–4302.
14. Kapsalis, C., B. Wang, …, C. Pliotas. 2019. Allosteric activation of an ion channel triggered by modification of mechanosensitive nano-pockets. *Nat. Commun.* 10:4619.
15. Chang, G., R. H. Spencer, …, D. C. Rees. 1998. Structure of the MscL homolog from *Mycobacterium tuberculosis*: a gated mechanosensitive ion channel. *Science.* 282:2220–2226.
16. Li, J., J. Guo, …, Z. Liu. 2015. Mechanical coupling of the multiple structural elements of the large-conductance mechanosensitive channel during expansion. *Proc. Natl. Acad. Sci. USA.* 112:10726–10731.
17. Yoshimura, K., A. Batiza, …, C. Kung. 1999. Hydrophilicity of a single residue within MscL correlates with increased channel mechanosensitivity. *Biophys. J.* 77:1960–1972.

18. Iscla, I., G. Levin, …, P. Blount. 2004. Defining the physical gate of a mechanosensitive channel, MscL, by engineering metal-binding sites. *Biophys. J.* 87:3172–3180.

19. Perozo, E., A. Kloda, …, B. Martinac. 2001. Site-directed spin-labeling analysis of reconstituted Mscl in the closed state. *J. Gen. Physiol.* 118:193–206.

20. Perozo, E., D. M. Cortes, …, B. Martinac. 2002. Open channel structure of MscL and the gating mechanism of mechanosensitive channels. *Nature.* 418:942–948.

21. Maurer, J. A., D. E. Elmore, …, D. A. Dougherty. 2000. Comparing and contrasting *Escherichia coli* and *Mycobacterium tuberculosis* mechanosensitive channels (MscL). New gain of function mutations in the loop region. *J. Biol. Chem.* 275:22238–22244.

22. Betanzos, M., C. S. Chiang, …, S. Sukharev. 2002. A large iris-like expansion of a mechanosensitive channel protein induced by membrane tension. *Nat. Struct. Biol.* 9:704–710.

23. Corry, B., A. C. Hurst, …, B. Martinac. 2010. An improved open-channel structure of MscL determined from FRET confocal microscopy and simulation. *J. Gen. Physiol.* 136:483–494.

24. Konijnenberg, A., D. Yilmaz, …, A. Koçer. 2014. Global structural changes of an ion channel during its gating are followed by ion mobility mass spectrometry. *Proc. Natl. Acad. Sci. USA.* 111:17170–17175.

25. Sukharev, S., M. Betanzos, …, H. R. Guy. 2001. The gating mechanism of the large mechanosensitive channel MscL. *Nature.* 409:720–724.

26. Blount, P., and I. Iscla. 2020. Life with bacterial mechanosensitive channels, from discovery to physiology to pharmacological target. *Microbiol. Mol. Biol. Rev.* 84:e00055-19.

27. Iscla, I., R. Wray, …, P. Blount. 2015. Scanning MscL channels with targeted post-translational modifications for functional alterations. *PLoS One.* 10:e0137994.

28. Bavi, N., D. M. Cortes, …, B. Martinac. 2016. The role of MscL amphipathic N terminus indicates a blueprint for bilayer-mediated gating of mechanosensitive channels. *Nat. Commun.* 7:11984.

29. Moe, P. C., G. Levin, and P. Blount. 2000. Correlating a protein structure with function of a bacterial mechanosensitive channel. *J. Biol. Chem.* 275:31121–31127.

30. Zhong, D., and P. Blount. 2013. Phosphatidylinositol is crucial for the mechanosensitivity of *Mycobacterium tuberculosis* MscL. *Biochemistry.* 52:5415–5420.

31. Powl, A. M., J. M. East, and A. G. Lee. 2003. Lipid-protein interactions studied by introduction of a tryptophan residue: the mechanosensitive channel MscL. *Biochemistry.* 42:14306–14317.

32. Reading, E., T. A. Walton, …, C. V. Robinson. 2015. The effect of detergent, temperature, and lipid on the oligomeric state of MscL constructs: insights from mass spectrometry. *Chem. Biol.* 22:593–603.

33. Liu, Z., C. S. Gandhi, and D. C. Rees. 2009. Structure of a tetrameric MscL in an expanded intermediate state. *Nature.* 461:120–124.

34. Powl, A. M., J. M. East, and A. G. Lee. 2008. Anionic phospholipids affect the rate and extent of flux through the mechanosensitive channel of large conductance MscL. *Biochemistry.* 47:4317–4328.

35. Powl, A. M., J. M. East, and A. G. Lee. 2007. Different effects of lipid chain length on the two sides of a membrane and the lipid annulus of MscL. *Biophys. J.* 93:113–122.

36. Laganowsky, A., E. Reading, …, C. V. Robinson. 2014. Membrane proteins bind lipids selectively to modulate their structure and function. *Nature.* 510:172–175.

37. Ward, R., C. Pliotas, …, O. Schiemann. 2014. Probing the structure of the mechanosensitive channel of small conductance in lipid bilayers with pulsed electron-electron double resonance. *Biophys. J.* 106:834–842.

38. Pliotas, C., R. Ward, …, J. H. Naismith. 2012. Conformational state of the MscS mechanosensitive channel in solution revealed by pulsed electron-electron double resonance (PELDOR) spectroscopy. *Proc. Natl. Acad. Sci. USA.* 109:E2675–E2682.

39. Pliotas, C., A. C. Dahl, …, J. H. Naismith. 2015. The role of lipids in mechanosensation. *Nat. Struct. Mol. Biol.* 22:991–998.

40. Rasmussen, T., V. J. Flegler, …, B. Böttcher. 2019. Structure of the mechanosensitive channel MscS embedded in the membrane bilayer. *J. Mol. Biol.* 431:3081–3090.

41. Rasmussen, T., A. Rasmussen, …, I. R. Booth. 2019. Interaction of the mechanosensitive channel, MscS, with the membrane bilayer through lipid intercalation into grooves and pockets. *J. Mol. Biol.* 431:3339–3352.

42. Saotome, K., S. E. Murthy, …, A. B. Ward. 2018. Structure of the mechanically activated ion channel Piezo1. *Nature.* 554:481–486.

43. Pliotas, C., and J. H. Naismith. 2017. Spectator no more, the role of the membrane in regulating ion channel function. *Curr. Opin. Struct. Biol.* 45:59–66.

44. Gao, Y., E. Cao, …, Y. Cheng. 2016. TRPV1 structures in nanodiscs reveal mechanisms of ligand and lipid action. *Nature.* 534:347–351.

45. Aryal, P., V. Jarerattanachat, …, S. J. Tucker. 2017. Bilayer-mediated structural transitions control mechanosensitivity of the TREK-2 K2P channel. *Structure.* 25:708–718.e2.

46. Erdogmus, S., U. Storch, …, M. Mederos Y Schnitzler. 2019. Helix 8 is the essential structural motif of mechanosensitive GPCRs. *Nat. Commun.* 10:5784.

47. Borbiro, I., D. Badheka, and T. Rohacs. 2015. Activation of TRPV1 channels inhibits mechanosensitive Piezo channel activity by depleting membrane phosphoinositides. *Sci. Signal.* 8:ra15.

48. De Vecchis, D., D. J. Beech, and A. C. Kalli. 2019. Molecular principles of Piezo1 activation by increased membrane tension. *bioRxiv* https://doi.org/10.1101/823518.

49. Buyan, A., C. D. Cox, …, B. Corry. 2019. Piezo1 induces local curvature in a mammalian membrane and forms specific protein-lipid interactions. *bioRxiv* https://doi.org/10.1101/787531.

50. Syeda, R., M. N. Florendo, and A. Patapoutian. 2016. Piezo1 channels are inherently mechanosensitive. *Cell Rep.* 17:1739–1746.

51. Teng, J., S. Loukin, …, C. Kung. 2015. The force-from-lipid (FFL) principle of mechanosensitivity, at large and in elements. *Pflugers Arch.* 467:27–37.

52. Martinac, B., J. Adler, and C. Kung. 1990. Mechanosensitive ion channels of *E. coli* activated by amphipaths. *Nature.* 348:261–263.

53. Malcolm, H. R., P. Blount, and J. A. Maurer. 2015. The mechanosensitive channel of small conductance (MscS) functions as a Jack-in-the box. *Biochim. Biophys. Acta.* 1848:159–166.

54. Reddy, B., N. Bavi, …, E. Perozo. 2019. Molecular basis of force-from-lipids gating in the mechanosensitive channel MscS. *eLife.* 8:e50486.

55. Wang, W., S. S. Black, …, I. R. Booth. 2008. The structure of an open form of an *E. coli* mechanosensitive channel at 3.45 A resolution. *Science.* 321:1179–1183.

56. Bass, R. B., P. Strop, …, D. C. Rees. 2002. Crystal structure of *Escherichia coli* MscS, a voltage-modulated and mechanosensitive channel. *Science.* 298:1582–1587.

57. Zhang, X., J. Wang, …, M. Yang. 2012. Structure and molecular mechanism of an anion-selective mechanosensitive channel of small conductance. *Proc. Natl. Acad. Sci. USA.* 109:18180–18185.

58. Lai, J. Y., Y. S. Poon, …, D. C. Rees. 2013. Open and shut: crystal structures of the dodecylmaltoside solubilized mechanosensitive channel of small conductance from *Escherichia coli* and *Helicobacter pylori* at 4.4 Å and 4.1 Å resolutions. *Protein Sci.* 22:502–509.

59. Anishkin, A., B. Akitake, and S. Sukharev. 2008. Characterization of the resting MscS: modeling and analysis of the closed bacterial mechanosensitive channel of small conductance. *Biophys. J.* 94:1252–1266.

60. Pliotas, C. 2017. Ion channel conformation and oligomerization assessment by site-directed spin labeling and pulsed-EPR. *Methods Enzymol.* 594:203–242.

61. Jeschke, G. 2012. DEER distance measurements on proteins. *Annu. Rev. Phys. Chem.* 63:419–446.

62. Schiemann, O., and T. F. Prisner. 2007. Long-range distance determinations in biomacromolecules by EPR spectroscopy. *Q. Rev. Biophys.* 40:1–53.

63. Ackermann, K., and B. E. Bode. 2018. Pulse EPR distance measurements to study multimers and multimerisation. *Mol. Phys.* 116:1513–1521.

64. Dürr, K. L., L. Chen, …, E. Gouaux. 2014. Structure and dynamics of AMPA receptor GluA2 in resting, pre-open, and desensitized states. *Cell.* 158:778–792.

65. Verhalen, B., R. Dastvan, …, H. S. Mchaourab. 2017. Energy transduction and alternating access of the mammalian ABC transporter P-glycoprotein. *Nature.* 543:738–741.

66. Bountra, K., G. Hagelueken, …, K. Beis. 2017. Structural basis for antibacterial peptide self-immunity by the bacterial ABC transporter McjD. *EMBO J.* 36:3062–3079.

67. Timachi, M. H., C. A. Hutter, …, E. Bordignon. 2017. Exploring conformational equilibria of a heterodimeric ABC transporter. *eLife.* 6:e20236.

68. Wingler, L. M., M. Elgeti, …, R. J. Lefkowitz. 2019. Angiotensin analogs with divergent bias stabilize distinct receptor conformations. *Cell.* 176:468–478.e11.

69. Michou, M., C. Kapsalis, …, G. Skretas. 2019. Optimization of recombinant membrane protein production in the engineered *Escherichia coli* strains SuptoxD and SuptoxR. *ACS Synth. Biol.* 8:1631–1641.

70. Ackermann, K., C. Pliotas, …, B. E. Bode. 2017. Sparse labeling PELDOR spectroscopy on multimeric mechanosensitive membrane channels. *Biophys. J.* 113:1968–1978.

71. Yardeni, E. H., T. Bahrenberg, …, D. Goldfarb. 2019. Probing the solution structure of the *E. coli* multidrug transporter MdfA using DEER distance measurements with nitroxide and Gd(III) spin labels. *Sci. Rep.* 9:12528.

72. Sahu, I. D., G. Dixit, …, G. A. Lorigan. 2020. Characterization of the human KCNQ1 voltage sensing domain (VSD) in lipodisq nanoparticles for electron paramagnetic resonance (EPR) spectroscopic studies of membrane proteins. *J. Phys. Chem. B.* 124:2331–2342.

73. Cieslak, J. A., P. J. Focia, and A. Gross. 2010. Electron spin-echo envelope modulation (ESEEM) reveals water and phosphate interactions with the KcsA potassium channel. *Biochemistry.* 49:1486–1494.

74. Deligiannakis, Y., and A. W. Rutherford. 2001. Electron spin echo envelope modulation spectroscopy in photosystem I. *Biochim. Biophys. Acta.* 1507:226–246.

75. Liu, L., D. J. Mayo, …, G. A. Lorigan. 2015. Determining the secondary structure of membrane proteins and peptides via electron spin echo envelope modulation (ESEEM) spectroscopy. *Methods Enzymol.* 564:289–313.

76. Liu, H., and J. H. Naismith. 2008. An efficient one-step site-directed deletion, insertion, single and multiple-site plasmid mutagenesis protocol. *BMC Biotechnol.* 8:91.

77. Branigan, E., C. Pliotas, …, J. H. Naismith. 2013. Quantification of free cysteines in membrane and soluble proteins using a fluorescent dye and thermal unfolding. *Nat. Protoc.* 8:2090–2097.

78. Denisov, I. G., Y. V. Grinkova, …, S. G. Sligar. 2004. Directed self-assembly of monodisperse phospholipid bilayer Nanodiscs with controlled size. *J. Am. Chem. Soc.* 126:3477–3487.

79. Hagelueken, G., R. Ward, …, O. Schiemann. 2012. MtsslWizard: in silico spin-labeling and generation of distance distributions in PyMOL. *Appl. Magn. Reson.* 42:377–391.

80. Martin, R. E., M. Pannier, …, H. W. Spiess. 1998. Determination of end-to-end distances in a series of TEMPO diradicals of up to 2.8 nm length with a new four-pulse double electron electron resonance experiment. *Angew. Chem. Int.Engl.* 37:2833–2837.

81. Jeschke, G., V. Chechik, …, H. Jung. 2006. DeerAnalysis2006—a comprehensive software package for analyzing pulsed ELDOR data. *Appl. Magn. Reson.* 30:473–498.

82. Chiang, Y. W., P. P. Borbat, and J. H. Freed. 2005. The determination of pair distance distributions by pulsed ESR using Tikhonov regularization. *J. Magn. Reson.* 172:279–295.

83. Giannoulis, A., R. Ward, …, B. E. Bode. 2013. PELDOR in rotationally symmetric homo-oligomers. *Mol. Phys.* 111:2845–2854.

84. von Hagens, T., Y. Polyhach, …, G. Jeschke. 2013. Suppression of ghost distances in multiple-spin double electron-electron resonance. *Phys. Chem. Chem. Phys.* 15:5854–5866.

85. Valera, S., K. Ackermann, …, B. E. Bode. 2016. Accurate extraction of nanometer distances in multimers by pulse EPR. *Chemistry.* 22:4700–4703.

86. Cutsail, G. E., III, J. Telser, and B. M. Hoffman. 2015. Advanced paramagnetic resonance spectroscopies of iron-sulfur proteins: electron nuclear double resonance (ENDOR) and electron spin echo envelope modulation (ESEEM). *Biochim. Biophys. Acta.* 1853:1370–1394.

87. Folgering, J. H., P. C. Moe, …, B. Poolman. 2005. Lactococcus lactis uses MscL as its principal mechanosensitive channel. *J. Biol. Chem.* 280:8784–8792.

88. Joseph, B., E. A. Jaumann, …, D. S. Cafiso. 2019. In situ observation of conformational dynamics and protein ligand-substrate interactions in outer-membrane proteins with DEER/PELDOR spectroscopy. *Nat. Protoc.* 14:2344–2369.

89. Joseph, B., A. Sikora, and D. S. Cafiso. 2016. Ligand induced conformational changes of a membrane transporter in *E. coli* cells observed with DEER/PELDOR. *J. Am. Chem. Soc.* 138:1844–1847.

Biophysical Journal, Volume *119*

Supplemental Information

In-Lipid Structure of Pressure-Sensitive Domains Hints Mechanosensitive Channel Functional Diversity

Charalampos Kapsalis, Yue Ma, Bela E. Bode, and Christos Pliotas

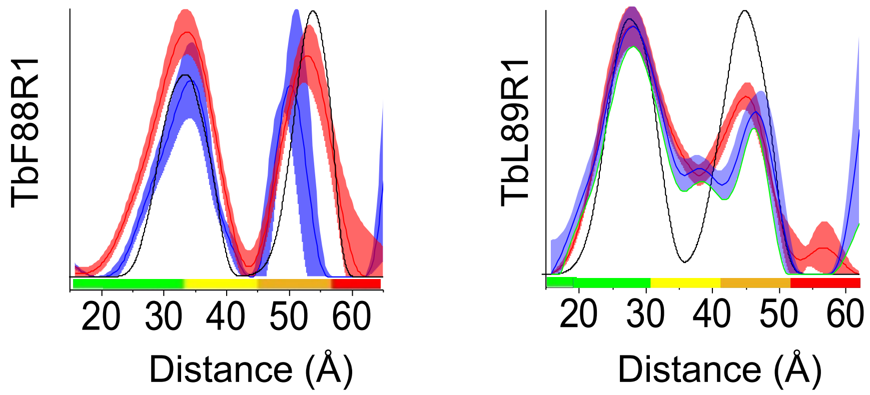

Fig. S1: Liposome (*E coli* polar extract) (shaded blue) and NDs (DMPC) (shaded red) PELDOR distance distributions compared to the modelled distances simulated from the x-ray (closed state) crystal structure (PDB 2OAR) using MtsslWizard (black line) for TbMscL F88R1 and L89R1.

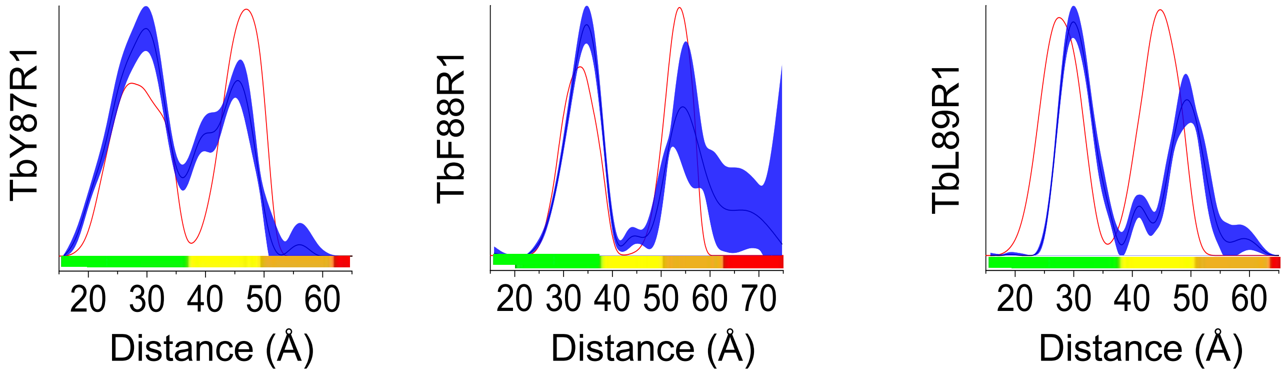

Fig. S2: PELDOR distance distributions of TbMscL in DDM (shaded blue) compared to the modelled distances simulated from the x-ray (closed state) crystal structure (PDB 2OAR) with MtsslWizard (red line).

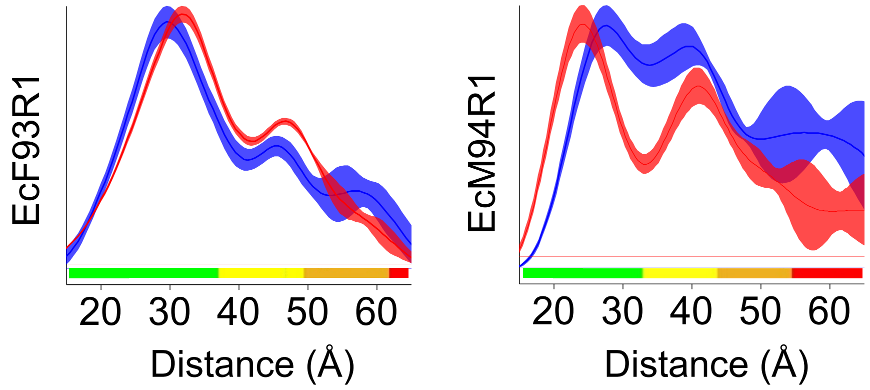

Fig. S3: EcMscL mutants PELDOR distance distribution comparison in DDM detergent (shade blue) and in nanodiscs (NDs) (shaded red).

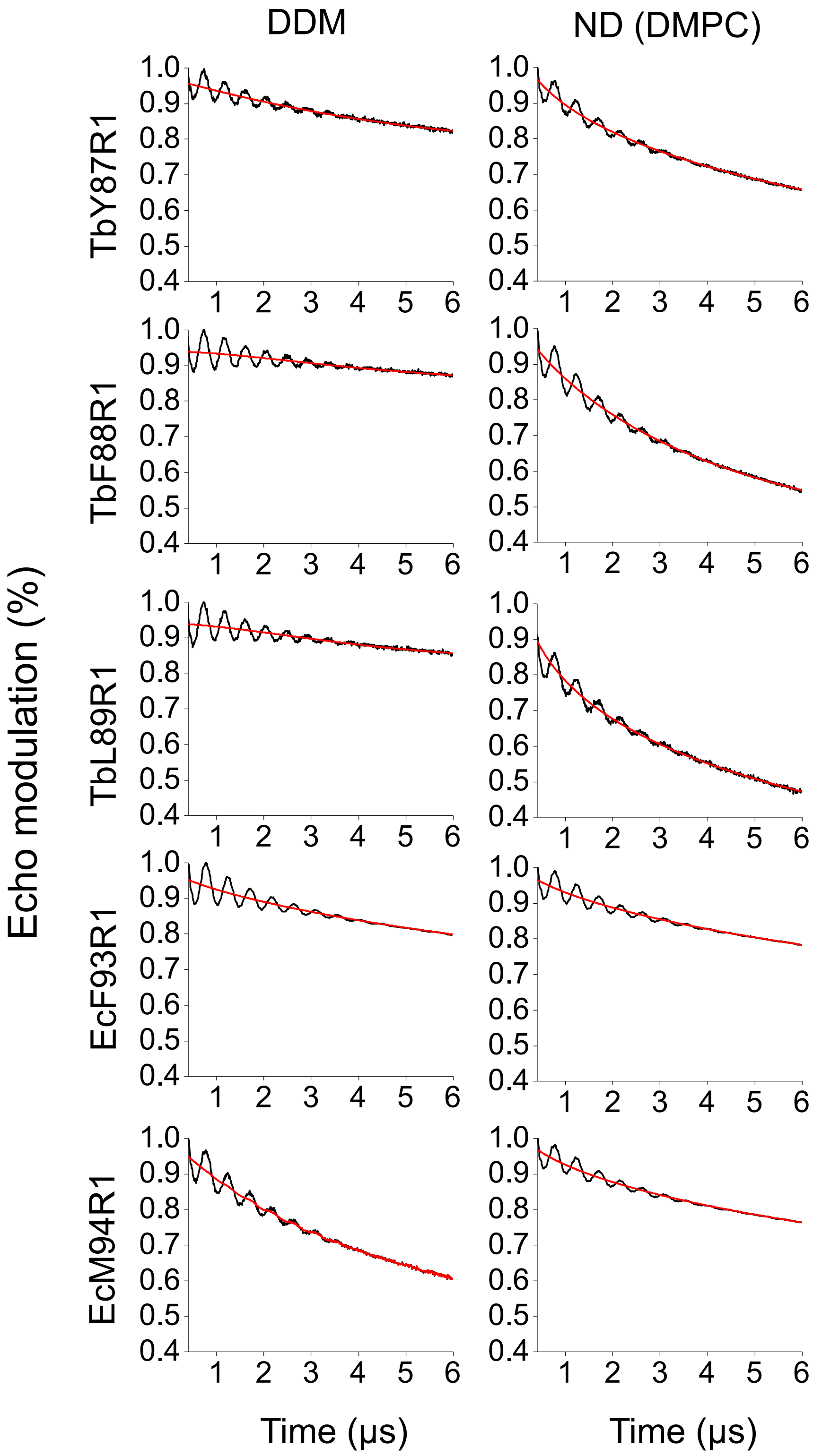

Fig. S4: Raw 3p-ESEEM data (black lines) and stretched exponential background fits (red lines).

		1	2	3	4	5
M. tuberculosis	1		29.45	21.66	29.75	68.99
E. coli	2	39		21.99	47.45	25.31
M. acetivorans	3	62	45		30.77	20.99
S. aureus	4	45	18	39		28.03
M. leprae	5	11	34	69	40	

Table S1: Pairwise alignment comparison between MscL orthologues. % sequence identity is shown in the upper/right half of the table and sequence residue gaps are shown in the lower/left half.

a.

Mutant	D_1; D_2; D_2/D_1 in DDM (Å)	D_1; D_2; D_2/D_1 in NDs (Å)
TbY87R1	29.7; 45.6; 1.55	26.3; 41.1; 1.57
TbF88R1	34.1; 55.8; 1.64	33.6; 53.1; 1.58
TbL89R1	30.0; 48.8; 1.63	27.8; 45.4; 1.63
EcF93R1	29.6; 45.8; 1.55	31.8; 46.9; 1.47
EcM94R1	27.7; 39.1; 1.41	24.1; 40.7; 1.69

b.

D_1 (TbL) – D_1 (EcL)	DDM (Å)	NDs (Å)
TbY87R1 - EcF93R1	0.1	-5.5
TbF88R1 - EcF93R1	4.5	1.8
TbF88R1 - EcM94R1	6.4	9.5
TbL89R1 - EcM94R1	2.3	3.7

Table S2: a. D_1 and D_2 PELDOR distance for each mutant in DDM and in nanodiscs (NDs) and their ratio. The expected ratios for symmetric multimers are 1.41 for a tetramer, 1.62 for a pentamer and 1.73 for a hexamer **b.** D_1 distance differences between distinct mutants for the two orthologues.

Mutant	% change in ^2H accessibility
TbY87R1	(23±5)%
TbF88R1	(28±4)%
TbL89R1	(32±7)%
EcF93R1	-(27±4)%
EcM94R1	-(19±4)%

Table S3: Percentage (%) change in deuterium (solvent) accessibility of MscL in detergent (DDM) following reconstitution in Nanodiscs (DMPC).
